# High hydrostatic pressure adaptive strategies in an obligate piezophile *Pyrococcus yayanosii*

**DOI:** 10.1038/srep27289

**Published:** 2016-06-02

**Authors:** Grégoire Michoud, Mohamed Jebbar

**Affiliations:** 1 Univ Brest, CNRS, Ifremer, UMR 6197-Laboratoire de Microbiologie des Environnements Extrêmes (LM2E), Institut Universitaire Européen de la Mer (IUEM), rue Dumont d’Urville, 29 280 Plouzané, France

## Abstract

*Pyrococcus yayanosii* CH1, as the first and only obligate piezophilic hyperthermophilic microorganism discovered to date, extends the physical and chemical limits of life on Earth. It was isolated from the Ashadze hydrothermal vent at 4,100 m depth. Multi-omics analyses were performed to study the mechanisms used by the cell to cope with high hydrostatic pressure variations. *In silico* analyses showed that the *P. yayanosii* genome is highly adapted to its harsh environment, with a loss of aromatic amino acid biosynthesis pathways and the high constitutive expression of the energy metabolism compared with other non-obligate piezophilic *Pyrococcus* species. Differential proteomics and transcriptomics analyses identified key hydrostatic pressure-responsive genes involved in translation, chemotaxis, energy metabolism (hydrogenases and formate metabolism) and Clustered Regularly Interspaced Short Palindromic Repeats sequences associated with Cellular apoptosis susceptibility proteins.

Deep sea hydrothermal vents are considered as oases of life and are characterized by a lack of sunlight, very high temperature (up to 460 °C) and high hydrostatic pressures (HHP)^1^. HHP represents a major parameter in the deep oceans, as the average hydrostatic pressure is estimated at 38 MPa. Pressure-adapted microbes or piezophiles are defined as organisms with an optimal growth rate at a pressure higher than atmospheric pressure (0.1 MPa). However, the effects of HHP on microbial physiology have mainly been studied on mesophilic piezosensitive bacteria (*e.g. Escherichia coli*) and pyschrophilic piezotolerant/piezophile microorganisms (*e.g. Photobacterium profundum* SS9)^2^. Only a handful of piezo-thermophiles have been studied, mainly belonging to the *Archaea* domain (*e.g. Thermococcus barophilus* MP)^3^,^4^. The most HHP-impacted cell component is the cytoplasmic membrane, which tends to become rigid due to the compaction of its lipid constituents in piezosensitive *Bacteria*^1^. These effects are counteracted in piezophiles by altering the membrane composition, especially the amount of monounsaturated fatty acids in lipids^5^; as an example, in *P. profundum* SS9 a piezophilic bacterium, the ratio of unsaturated/saturated membrane lipids increases the membrane fluidity under HHP^6^. A lot of processes like transporters, and motility and respiratory chain components, directly related to the membrane composition are also affected by HHP in piezophiles^5^. The regulation of the genes and enzymes involved in the respiratory chain of several piezophiles is particularly interesting because it illustrates that these microorganisms have developed differential mechanisms to counteract the effect of HHP. As an example, the piezophilic *Shewanella violacea* possesses two cytochrome protein complexes (c and d) that are differentially regulated under HHP, suggesting a shift in the electron chain and the respiration system even though oxygen consumption remains unchanged^7^. These two examples show that the effects of HHP on piezophiles are not due to a specific group of enzymes or genes but to an overall switch of the cell metabolism, not as a stress response *per se* but more as a fine tuning adaptation to deep life conditions. This kind of adaptive strategy is further supported by two recent studies on piezophiles, *T. barophilus* and *Desulfovibrio hydrothermalis*^4^,^8^, where authors showed that piezophilic adaptation goes through major cellular processes, such as the transport of solutes like amino acids, amino acid metabolism and pathways involved in energy production. Since nearly all studies on pressure adaptation were done on piezophiles or piezotolerants, all capable of growing at atmospheric pressure, it is possible that these strains have evolved a dual strategy to cope with both HHP and low pressure. In fact, very little is known about the HHP adaptive mechanisms of the few obligate piezophile organisms isolated up to date. Among these, *Pyrococcus yayanosii* sp. nov., is the first and only known obligate piezophilic hyperthermophilic archaeon, and was isolated from one of the deepest vent fields yet explored^9^,^10^. This species was therefore chosen as the model organism to study the effects of HHP from a genomic, physiological, proteomic and transcriptomic point of view, in comparison to *Pyrococcus* species, which are not obligate piezophiles. Optimal (52 MPa) and stressful pressures (20 and 80 MPa) were experimentally determined for *P. yayanosii*. Here we show, using transcriptomic and proteomic analyses, the importance of several gene clusters and metabolic pathways, such as energy production and conversion, in the adaptation to HHP, and provide new insight into the piezophilic lifestyle of hyperthermophilic microorganisms.

## Results and Discussion

### General features of the *P. yayanosii* CH1 genome compared with other *Pyrococcus* species genomes

**Figure 1:**
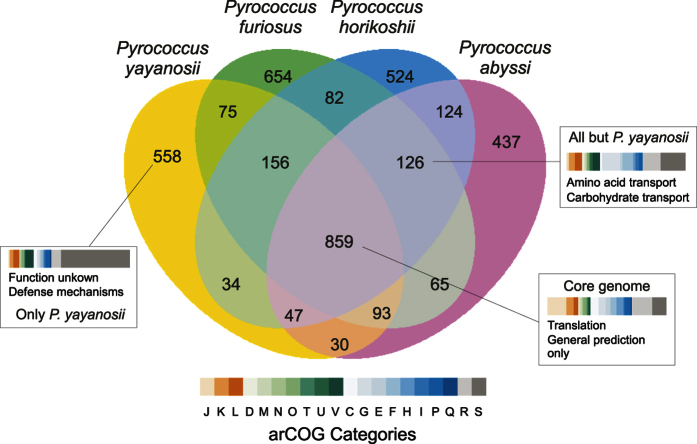
Four-way Venn diagram of orthologous genes calculated by reciprocal hits. *P. yayanosii* (yellow), *P. furiosus* (green), *P. horikoshii* (blue) and *P. abyssi* (purple). Numbers shown in the Venn diagram correspond to the orthologous gene groups given in the respective patterns. arCOG categories represent the information storage and processing (orange palette), cellular processes and signaling (green palette), metabolism (blue palette) and poorly characterized genes (grey palette).

We first compared the genomic features of *P. yayanosii* CH1 with three other *Pyrococcus* isolates: *P. furiosus* DSM 3638, *P. abyssi* GE5 and *P. horikoschii* OT3 (Table S1). These isolates were chosen because their physiological characteristics are similar to *P. yayanosii* (Optimal temperature, salinity, pH, etc.) with the exception of their isolation depth and consequently their piezophily (Table S1). Indeed, *P. furiosus* and *P. horikoshii* are both piezosensitive, *P. abyssi* is a piezophile and *P. yayanosii*, as stated earlier, is an obligate piezophile^9^. Their genomic features are similar, with the exception of the genome size that is 200 kb larger for *P. furiosus* (1.9 Mb vs. 1.75 Mb) and of the GC% that is higher for *P. yayanosii* (52% vs 40–45%)^11^. As *P. yayanosii* is the only obligate piezophile among the *Pyrococci*, a comparison was made to try to find piezophilic markers. Core genomes, pan-genomes and unique genes were thus determined for each strain using the EDGAR server^12^ (Fig. 1). This comparative analysis, based on groups of orthologues, revealed 859 common orthologous proteins shared by all four species (Fig. 1), representing a little more than half of the proteins in each strain (39–45%). These proteins are preferentially involved in translation and ribosomal structure (J) and have a general functional prediction only (R). Of the unique genes of *P. yayanosii,* more than 60% are currently uncharacterized, and the genes involved in defense mechanisms are overrepresented. The proteins involved in defense mechanisms are almost all CRISPR (Clustered Regularly Interspaced Short Palindromic Repeats) -associated proteins. As CRISPRs play a role in the prokaryotic immune system^13^, it has been suggested that their presence is correlated with infections by mobile genetic elements (viruses and plasmids), even though proviruses and plasmid(s) are not present in the *P. yayanosii* genome. The overrepresentation of these sequences is probably due to the high plasticity of these structures and is illustrated by the fact that two *P. yayanosii* CRISPR/*cas* clusters out of four are not found in the other *Pyrococcus* species (Figure S1).

A comparison between all species except *P. yayanosii* shows that genes involved in amino acid, carbohydrate biosynthesis and transport are over-represented. Regarding the predicted amino acid pathways for the four strains, *P. yayanosii* appears to lack several important biosynthesis pathways, such as the basic and aromatic amino acid pathways (Table S2). The fact that the aromatic amino acid biosynthesis pathway, and especially tryptophan, are missing from *P. yayanosii* is in agreement with the downregulation of a similar pathway in the piezophile bacterium *D. hydrothermalis* when placed under high pressure^8^. As tryptophan is the largest amino acid and its synthesis has a high energy cost in bacteria^14^, one may think that the import may be more favorable than *de novo* synthesis. In support, Cario and colleagues (2015) showed that the requirement for amino acids increases from 3 to 17 at high pressure in the piezophilic *T. barophilus*^3^.

In order to determine the highly expressed genes in each genome, Codon Adaptation Indices (CAI) were calculated using ribosomal proteins as references^15^. The CAI takes into account the fact that highly expressed genes show a strong bias for particular codons^16^. Excluding the reference genes, the first hundred genes that had the highest CAI were then studied (Figure S2). As could be expected according to the literature, the genes involved in information storage and processing were overrepresented (20%)^17^. Concerning *P. yayanosii*, genes involved in energy production and conversion (C) were found nearly twice as often (17%) as in other strains (9–11%). Among these, we find genes involved in ATP/ADP synthase (V-ATPase) and also genes coding for hydrogenases and ferredoxin oxydoreductases. In *Pyrococcus*, hydrogenases are involved in a simple respiratory system where they link the oxidation of ferredoxin with the formation of hydrogen and store energy in the form of an ion gradient^18^. These results suggest that *P. yayanosii* implemented a seemingly highly expressed system in order to produce and conserve energy under HHP.

### Effect of hydrostatic pressure on the *P. yayanosii* transcriptome and proteome

We chose *P. yayanosii* as our study organism because it was the first hyperthermophilic obligate piezophile described^9^. Preliminary studies and our experimental results are in accordance with an optimal pressure of 50–52 MPa for this species, so we chose 52 MPa as our optimal pressure^10^. In order to determine stressful pressures, where growth rates are cut by half relative to performances at optimal pressure, growth kinetics were assessed at pressures ranging from 0.1 to 80 MPa (Figure S3). As a result, 20 and 80 MPa were selected as sub- and supra-optimal pressures, respectively, for *P. yayanosii*. Microarray and proteomics analyses were then performed as described in the methods section. Concerning the proteomics analysis, 794 proteins were encountered, which corresponded to 42% of the *P. yayanosii* proteome. These proportions are slightly higher than those described previously in a similar type of study done on *Thermococcus onnurineus*, in which it was grown on different carbon sources^19^. The proteins identified in this study have an average molecular weight of about 40 kDa, with a minimum of 4 and a maximum at 200 kDa, which approximately corresponds to the whole proteome.

**Figure 2:**
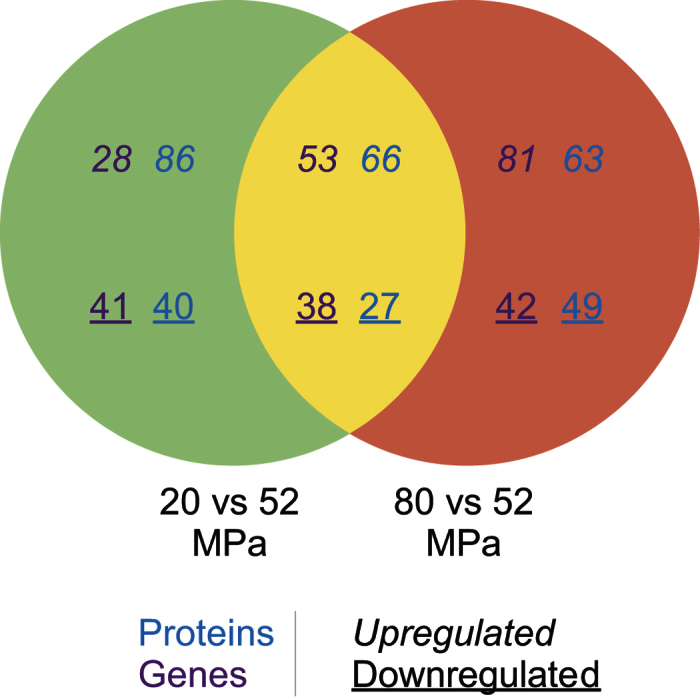
Venn diagram of protein and gene regulation in *P. yayanosii*. Genes are shown in purple, and proteins in blue. Upregulated elements are marked in italics and downregulated elements in underlined.

*P. yayanosii* multi-omics analysis showed that 81 genes and 152 proteins were upregulated at 20 MPa compared with 52 MPa, whereas 79 genes and 67 proteins were downregulated. At 80 MPa compared with 52 MPa, 134 genes and 129 proteins were upregulated, and 80 genes and 76 proteins were downregulated. When comparing both 20 and 80 with 52 MPa, one can see that 53 genes and 66 proteins were upregulated, whereas 38 genes and 27 proteins were downregulated in both conditions (Fig. 2 and Table S3).

Real-time RT-PCR was used to validate the transcriptomic data of a set of 22 selected genes encoding hydrogenase subunits, regulators, transporters, Cas proteins and conserved hypothetical proteins. As illustrated in Table S4, their expression changes revealed by real-time RT-PCR resulted in more highly variable results than those from microarrays, illustrating the larger dynamic range of this method compared with microarray measurements^20^.

Enriched pathways were determined using KEGG annotations and GO ontology, which showed distinctive regulations between the transcriptome and proteome. Genes involved in energy metabolism via hydrogenases were downregulated in sub-optimal, as well as supra-optimal pressure conditions, whereas the genes involved in chemotaxis, translation via the ribosomes and genes coding for transmembrane ATPases were upregulated under stressful conditions. Concerning the translational regulations, proteins involved in translation and formate metabolism were downregulated, whereas those involved in tRNA biosynthesis, hydrogenases and proteins that possess an oxidoreductase activity were upregulated under stressful pressure conditions (Table 1).

### Hydrostatic pressure impacts the chemotaxis pathway

*P. yayanosii* genes coding for the chemotaxis pathway (PYCH_15450–490) were all upregulated in a transcriptional manner at 20 and 80 MPa compared with the 52 MPa reference. These genes encode CheA, two CheC, CheD and a methyl-accepting chemotaxis protein (MCP). The MCP acts as the stimulus receptor and permits autophosphorylation of the histidine kinase CheA. The phosphate is then used as a substrate for the CheY protein. The CheC protein then hydrolyses CheY and is connected to the archaellum by a mechanism that is still unknown^21^. Two modes of swimming exist one where the flagella or archaellum rotates in a counterclockwise direction and the cell moves forward, and one where the mobility apparatus rotates in a clockwise direction and the cell changes direction. In *E. coli*, clockwise rotation is caused by binding of the phosphorylated response regulator CheY. In this piezosensitive bacterium, HHP seems to have the same effect on the mobility, causing a tumbling of the cell^22^. The overexpression of this cluster in *P. yayanosii* could similarly indicate an increasing number of clockwise rotations of the archaellum, and thus an increase in tumbling. As a hypothesis, this regulatory response could facilitate the search for nutrients under stressful conditions. This effect is amplified by increased amino acid requirements of the cell, as observed at high pressure in a piezophilic archaeon^3^.

### Effect of hydrostatic pressure on translation machinery

**Figure 3:**
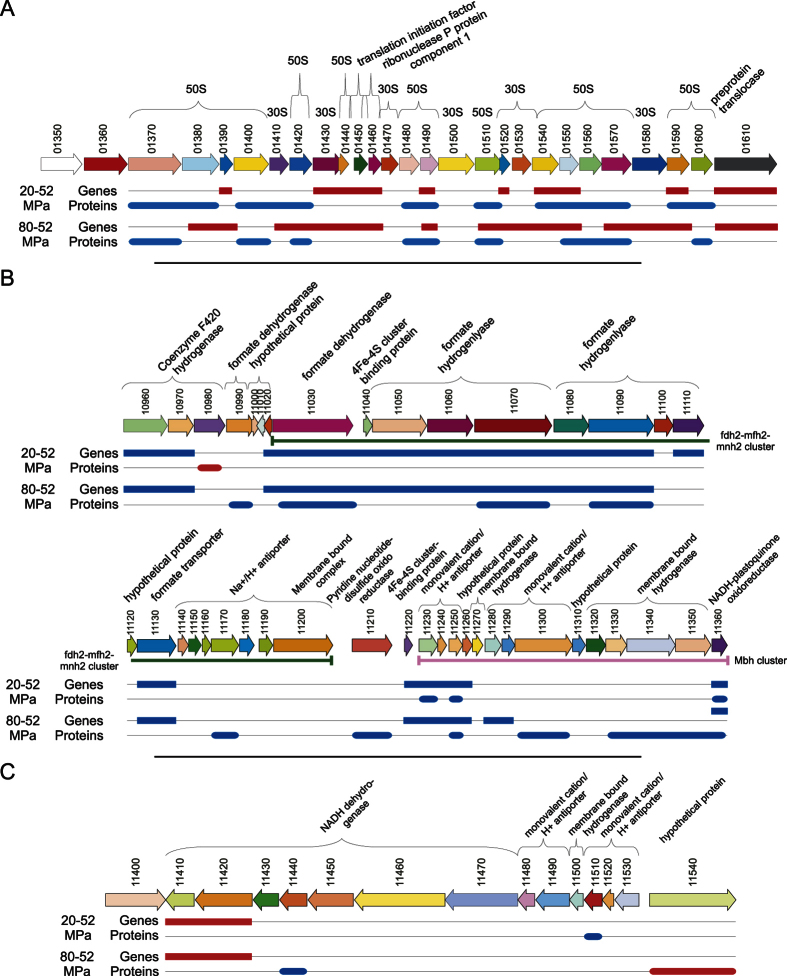
Regulated clusters under low and high pressure. (**A**) Ribosomal clusters, (**B**) Formate and hydrogenase clusters, (**C**) Sulfhydrogenase cluster. The arrows represent the genes, the ovals the RNA expression and the rectangles the protein expression results. Red indicates upregulation and blue indicates downregulation on conditions given on the left. The green bar corresponds to the cluster fdh2-mfh2-mnh2 that was described in *T. onnurineus* as responsible for formate metabolism^29^. The purple bar corresponds to the Mbh cluster that was described in *P. furiosus* as involved in the reduction of protons to H_2_^18^.

Ribosomal subunits were also regulated under conditions stressful for *P. yayanosii* (PYCH_01350–1610). Out of 24 genes, 10 and 16 were upregulated at 20 and 80 MPa, respectively, whereas 15 and 10 proteins were downregulated at low and high pressure (Fig. 3). Among the 24 aminoacyl-tRNA synthetase proteins that *P. yayanosii* possesses, 15 are translationally up-regulated under both low and high pressure; three genes are also upregulated under the same conditions. Upregulation of tRNA synthetases suggests an increased need for tRNA in translation. The activity of these enzymes under HHP has only been measured in *E. coli*, a piezosensitive bacterium, where it decreases sharply above 10 MPa^23^. The opposite regulation of ribosomal subunits from a transcriptional and translational point of view at both low and high pressure can either be due to the disrupting effects of low and high pressure or to the starvation of the cell under stressful conditions. The overexpression of the tRNA synthetases suggests greater protein biosynthesis and enzymatic activity under stressful pressures, as an increased synthesis of ribosomal subunits could counteract the effects of HHP on the ribosome complex. This is further supported by the overexpression at both low and high pressure of the protein PYCH_12080, which encodes an ATPase (ABCE1) that plays a crucial role in ribosome recycling^24^.

### Hydrogenases and energy conversion

One of the main pathways regulated in *P. yayanosii* under stressful pressures is the energy pathway with hydrogenases, coupled with formate metabolism (Table 1). Hydrogenases are metalloproteins involved in the reaction 2H^+^ + 2e^−^ ↔ H_2_^25^. In *Thermococcales*, hydrogenases can be classified into different clusters, Mbh, SHI, SHII, Mbx^18^,^26^. These clusters were determined for *P. yayanosii* by homologies with *P. furiosus* genes^18^. The Mbh cluster in *P. yayanosii* corresponds to locus PYCH_11230–360. Out of the 13 genes that are part of this cluster, five and two were transcriptionally and translationally downregulated under low pressure, and seven mRNA and five proteins were also downregulated at 80 MPa (Fig. 3). The Mbx cluster (PYCH_11410–530, 13 genes) is not significantly regulated except for the PYCH_11410–20 genes, which are transcriptionally upregulated under both low and high pressure. Two proteins, PYCH_11440 and PYCH_11510, are downregulated under low and high pressure, respectively. Both the SHII (PYCH_08370–400) and the SHI cluster (PYCH_00010–40) are also regulated. The SHII cluster is downregulated transcriptionally (4/4) under low and high pressure and translationally (3/4) at high pressure. The SHI cluster is similarly downregulated transcriptionally (2/4) at 20 and 80 MPa, whereas one protein is upregulated at high pressure. The function of these last two clusters is still unclear in *P. furiosus* as their deletion doesn’t seem to have an effect on the strain. The proteins encoded by the Mbh cluster have been suggested to represent a simple form of respiration, in that they generate molecular H_2_ and can use the energy to create an ion gradient across the membrane^27^. Mbh and Mbx protein clusters are homologous and can accept electrons from ferredoxin to create an ion gradient. However, in *P. furiosus*, the main difference between the two is that Mbx is active in the presence of sulfur and couples the oxidation of ferredoxin to NADPH formation with the implication of an NADPH elemental sulfur reductase (NSR) and glutaredoxin-like protein (Pdo)^28^. The upregulation of part of the Mbx and SHII cluster probably accounts for the results observed in Table 1.

The main cluster regulated under pressure codes for formate metabolism (PYCH_11030–200)^29^,^30^. Among the 17 genes of this cluster, 10 and eight are downregulated transcriptionally at low and high pressure, respectively. Even though they are not translationally regulated at low pressure, some (3) are downregulated at high pressure (Fig. 3). In *Thermococcales*, formate is oxidized to CO_2_ by a formate dehydrogenase and then protons are reduced to H_2_ by a hydrogenase in response to formate accumulation during fermentation^29^,^31^. This allows the cells to maintain homeostasis of their cytoplasmic pH. Even though different formate clusters exist in *Thermococcales,* only one seems to be responsible for cell growth on formate in *T. onnurineus* (fdh2-mfh2-mnh2)^29^. This cluster shows strong homology with the *P. yayanosii* cluster, suggesting that *P. yayanosii* could metabolize formate. This has been confirmed recently in the mutant *P. yayanosii* strain A1, a derivative strain of CH1 that can grow at atmospheric pressure. Indeed, Li in 2014, showed that when this cluster was deleted, formate accumulation was detected with a loss of viability at atmospheric pressure^32^. Interestingly, this accumulation was also detected after the deletion of Mbh and SHI encoding genes using a recently developed genetic tool^33^, which indicated that they are involved in the formate metabolism. However, it seems that this strain is unable to grow solely on formate^32^.

Given the transcriptional downregulation of this cluster in low and high pressure, one could propose that in optimal conditions, the strain derives energy from the conversion of formate to bicarbonate and H_2_ to sustain growth, suggesting a difficulty for the cell to transport formate when placed in stressful conditions. In *P. furiosus*, when sulfur is added, the Mbh cluster is downregulated and replaced by the Mbx cluster, as shown in a transcriptomic analysis by Schut *et al.*^28^. Interestingly, part of this cluster is upregulated both transcriptionally and translationally in *P. yayanosii* under stressful conditions and, at the same time, the NSR (PYCH_07890) and Pdo proteins (PYCH_16300) are also upregulated at low and high pressure, as shown in Table 1. These results strongly suggest a pressure-induced energetic shift similar to what happens in *P. furiosus* on the addition of sulfur^18^.

All the genes coding for a transmembrane ATPase were transcriptionally upregulated at 80 MPa compared with 52 MPa (PYCH_15710–60). These genes are subunits of a V-ATPase that generates a proton gradient driving the accumulation of solutes^34^. Incidentally, it has been shown that acidification of the cytosol in yeasts due to high pressure has an impact on glycolysis^35^ and that an ATPase plays a role in removing the H^+^ ions to vacuoles, thus increasing the cytosolic pH^36^. One could then suppose that the overexpressed V-ATPase genes could regulate the intracellular pH under very HHP, or could counterbalance proton production by the Mbx.

### CRISPR/Cas system

Other regulated clusters in *P. yayanosii* included genes involved in replication and CRISPR-*cas*. Concerning replication, we found two loci (PYCH_06170 and PYCH_06190) coding for a bipolar DNA helicase and a rad50 ATPase. The PYCH_06170 locus is translationally upregulated at high pressure, whereas PYCH_06190 is translationally upregulated under the same conditions. This upregulation is correlated with the regulation of another cluster PYCH_16160–210 which is composed, among others, of the *cdc6* gene. Among the six loci of this cluster, three and five are translationally upregulated at low and high pressure, respectively. Its presence strongly suggests that the replication origin is situated in this cluster, which was confirmed by a Blast search done using the origin replication determined in *P. furiosus*^37^. The upregulation of these replication genes, along with the regulation of the ribosomal cluster, strongly suggests a stress response of *P. yayanosii* to HHP, whereas the regulation of hydrogenases and formate enzymes could be considered a metabolic shift and thus a metabolic adaptation.

*P. yayanosii* possesses three CRISPR-*cas* clusters, which are not regulated in the same way. Five and two *cas* genes from the PYCH_07940–8060 cluster are transcriptionnally downregulated at low and high pressure, respectively. Another regulated cluster is comprised of 14 genes, two of which are over-expressed at 20 and another two of which are over expressed at 80 MPa, respectively. The differential regulation of these clusters seems to support the hypothesis that these regions are subject to extensive rearrangements, even though the CRISPR sequences do not match those of known viruses. This is probably due to the fact that only two *Thermococcales* viruses have ever been sequenced^38^.

Another genomic cluster that is comprised of a CRISPR-*cas* system is also regulated under stressful pressure conditions (PYCH_14110–590) (Figure S4). Among this cluster, we find a region that seems to be an ancient integration of a genomic element, characterized by the presence of a SSVI integrase (PYCH_14110–300)^39^. Almost all the genes code for hypothetical proteins and 13 are transcriptionally up-regulated at low pressure (Figure S4). Also, two proteins responsible for the cyclic 2,3-diphospoglycerate (cDPG) synthesis (PYCH_14390–400) are upregulated at 20 and 80 MPa. The cDPG is a low molecular weight compound that accumulates to high levels in some hyperthermophilic methanogens like *Methanopyrus kandleri*. Studies suggest that it is involved in temperature adaptation or energy storage^40^,^41^. In *P. yayanosii*, cDPG could play a role in energy supply to cope with the constraints of HHP. The last part of the cluster corresponds to the PYCH_14430–460 genes coding for CRISPR-*cas* (Figure S4). Four loci are transcriptionally upregulated at low pressure, and seven are translationally downregulated at 80 MPa.

These results seem to suggest a particular role of *cas* genes in the resistance or sensitivity to high pressure, even though they are believed to confer resistance to foreign genetic elements such as viruses and plasmids^42^. However, a few studies done on *Bacteria* or fungi have indicated a potential role of *cas* genes in biofilm or spore formation^43^,^44^. One could then suggest that, in some cases, *cas* genes could play a role in transcriptional regulation in a similar way to the RNA interference system in eukaryotes^45^.

## Conclusion

**Figure 4:**
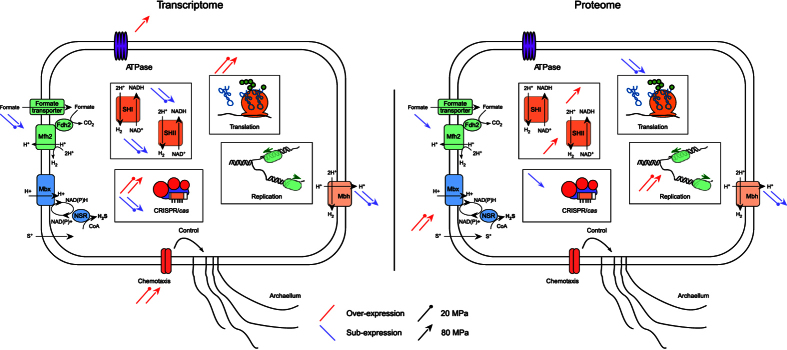
Schematic representation of the main regulations observed at low and high pressure. Here are represented the formate metabolism (green), Mbh Hydrogenase (light orange), Mbx sulfhydrogenase (blue), SHI and SHII sulfhydrogenase (dark orange), the chemotaxis system along the archaellum, CRISPR/*cas* system, membrane-bound ATPase and the translation and replication complexes. The transcriptomic regulation is presented on the left whereas the proteomic one is on the right side. Blue and red arrows represent the sub- and over-expression in stress conditions (20 or 80 MPa) compared to the optimum condition (52 MPa). The circle and arrow lines correspond to the regulation at respectively 20 and 80 MPa. The differences observed for the CRISPR/cas system is due to the opposite regulations of the different clusters.

Results described in this manuscript showed that the responses to high and low pressure are mostly equivalent except for certain functions like transmembrane ATPases and, replication (Fig. 4). Hydrostatic pressure also impacts the chemotaxis pathway, translation via ribosomes, CRISPR-*cas* and energy metabolism, with the regulation of hydrogenases, oxidoreductases and formate metabolism (Fig. 4). This last regulation constitutes a large metabolic shift, with the down-regulation of hydrogenases and formate metabolism and the up-regulation of oxidoreductases (Fig. 4).

Thus the H_2_ metabolism (Mbh, SHI/SHII and Mfh2 hydrogenases) is repressed at low and high pressure, while the energy could be supplied mainly by the sulfur-dependant hydrogenases (Mbx) activity, which are surexpressed under these conditions at translationally level (Fig. 4). This metabolic shift might be a result of the adaptive mechanism of cell membrane-embedded proteins to changing membrane fluidity as has been demonstrated on other piezophiles that showed major effects of high pressure on cellular membranes and energy metabolism^46^. The H_2_ and H_2_S production and the hydrogenases activities should be measured at optimal, sub- and supraoptimal HP to gain into insight of the regulation of the cells of their energy demand under large range of HP. One can conclude that the modulation of respiration pathways is one of the piezoadaptation mechanisms by which microorganisms adapt to variations in hydrostatic pressure. Chemotaxis machinery that depends on respiration activity and membrane potential is also impacted by low and high pressure at transcriptional level, which might be due too to change in membrane fluidity. The regulation of genes involved in replication and translation (ribosomal subunits) confirm the extensive effect of HHP on fundamental cell processes. The up-regulation of these genes implies a disruptive role of HHP on complex protein structure (ribosomes and the replication mechanism) related to nucleic acids. The next step in the study of piezophiles could be to perform several adapted protocols to analyze the metabolome which could potentially provide a more accurate picture of the true physiological state of the cell in response to hydrostatic pressure change^47^.

## Methods

### Media and growth conditions

*Pyrococcus yayanosii* strain CH1 was isolated from chimney samples harvested from the Ashadze hydrothermal site, at 4,100 m depth on the Mid-Atlantic ridge^9^,^10^ in March 2007. It was grown at 98 °C under anaerobic conditions in TRM medium (“*Thermococcales* Rich Medium”)^10^. High hydrostatic pressure cultures were grown in sterile syringes and incubated in a high hydrostatic pressure/high-temperature incubator (Top Industrie) from 0.1 MPa to 80 MPa, as previously described^48^. Once the middle of the exponential phase was reached (after 4 hours), the cells were slowly decompressed and immediately placed in previously frozen falcon tubes, in order to minimize the degradation of the cell component (RNA and proteins).

### Determination of cell numbers

Growth was monitored by cell counting using a Thoma chamber and photonic microscopy at x40 (Olympus) magnification or using flow cytometer (CyFlow Space, Partec). Cells were fixed with 2% paraformaldehyde and counted with one of the two previous described methods.

### DNA purification and extraction

Genomic DNA extraction of *P. yayanosii* CH1 was performed using a phenol–chloroform–isoamyl alcohol (PCI) method as described by Thiel *et al.*^49^. The DNA was quantified by NanoDrop and the quality of extraction was checked by electrophoresis on a 1% agarose gel containing ethidium bromide at a final concentration of 0.5 mg/ml (in a bath of 40 mM Tris pH 8, 40 mM acetate-1 mM EDTA pH 8 (TAE) 1X). The migration was performed at 85 V for 40 min, with a 1 kb ladder (Promega) as the size marker. The routine tests by PCR amplification were performed using Taq Polymerase (Promega).

### RNA purification and extraction

The total RNA extraction method has been described in Vannier *et al.*^4^. RNA samples (≈750 μg) were sent to the Imaxio company for microarray analysis, where the quality was assessed by Bioanalyzer 2100 (Agilent Technologies). Low Input Quick Amp WT Labeling Kit, One color (Agilent Technologies) was used to synthesize labeled cRNA from 50 ng of total RNA. The amplified cRNA were purified using Qiagen’s RNeasy Mini Kit, quantified by Nanodrop ND-1000 and analyzed by Agilent Bioanalyzer. The cRNA quantity ranged between 3 and 5 μg. cRNA were fragmented at 60 °C for 30 min in a specific buffer to obtain fragments between 50 and 200 nucleotides. Hybridization was performed using 600 ng cRNA at 65 °C for 17 h. After the hybridization, the slides were washed, and the microarrays were scanned using an Agilent G2505C scanner. Feature extraction and image analysis software (Feature Extraction Software; Agilent Technologies) was used to locate and delineate every spot in the array and to integrate each spot’s intensity, using the simple color Agilent protocol (GE1_1105_Oct12). The microarray results were deposited in the GEO platform of the NCBI (Accession number: GSE72783).

Normalization and statistical analysis were done by the platform “*Génomique Santé de Biosit*” in Rennes, France (http://ouestgenopuces.univ-rennes1.fr/). First, data were threshold (values below 1.0 were set equal to 1.0) and log2-transformed. Normalization was performed using per-array and per-gene median normalization methods (scaling). TMEV software^50^ was used to perform the differential expression analysis. Genes differentially expressed between conditions were identified using a multi-class SAM (Significant Analysis of Microarray). Adjusted p-values were calculated by controlling for a false discovery rate (FDR) of 5%.

### Microarray

The transcriptome was analyzed using an Agilent Microarray. It was designed using the Agilent eArray 5.0 program according to the manufacturer’s recommendations. Each customized microarray (8 × 60 K, AMADID 048498, Agilent Technologies) contains the whole genome covered by 60-mer oligonucleotides, representing the genes, intergenic space and non-coding RNA. The genes and intergenic space where extracted from the NCBI GenBank Database.

### Expression Profiling by Real-time RT-PCR

Total RNA and cDNA synthesis was performed as described above. PCR primer pairs were designed using Primer3 software^51^ with standard parameters and calibrated with genomic DNA. qPCR was done using the SYBR® Green PCR Master Mix it (Applied Biosystems). For one reaction, 12.5 μl of Master Mix was used, along with 1 μM of primers and 1 ng of cDNA. Standard conditions were used with the StepOnePlus™ Real-Time PCR System (Applied Biosystems), DNA denaturation at 95 °C for 10 min followed by 40 cycles of 95 °C for 10 s, 60 °C for 10 s and 72 °C for 15 s. Gene expression was calculated relative to the transcripts levels of the 16S rRNA gene using the formula ΔΔCt and the software supplied by the system.

### Protein extraction and analysis

Protein extraction was done as described by Lee *et al.*^52^ in quadruple for each condition. 100 ml of cells were centrifuged at 8,000 *× g*, for 15 min at 4 °C. The pellet was resuspended in 1 ml of TE buffer and recentrifuged. 500 μl of extraction buffer (25 mM Tris-HCl pH 8, 1% SDS and 5 mM DTT) was used to resuspend and vortex the cells for at least 2 min. The suspension was sonicated on ice at 60% amplitude using 2 s pulses in an Ultrasonic Processor (Misonix) for 20 pulses. Cellular debris was removed by centrifugation at 25,000 *× g* for 30 min at 4 °C. The whole cell lysate supernatant was incubated in a 37 °C water bath for 1 h to reduce completely the sample. Protein content was assessed with the 2D Quant Kit (GE Healthcare).

Spectral-counting based label-free quantitative proteomics was performed to identify differentially expressed proteins. First, short 1-D SDS-PAGE runs (5–12%) were performed with 10 μg of total proteins allowing the proteins to migrate for 5–7 mm in length. Gels were stained with Coomassie blue (BioRad), roughly cut and sent to PAPPSO platform facilities, Jouy-en-Josas, France. The analysis presented here were more thoroughly described in Arfi *et al.*^53^. Each gel was cut in a band of approximately 2 mm width and 5–7 mm length. Bands were then washed and digested with trypsin (Promega). Tryptic peptides were extracted, concentrated and desalted on a precolumn cartridge (300 μm i.d., 5 mm Dionex). A separating column (75 μm i.d., 150 mm, Nanoseparations) was used to eluate peptides. Peptides were analyzed with a Q-exactive mass spectrometer (Thermo Fisher Scientific, USA) using a nanoelectrospray ion source. The raw mass data were first converted to mzXML format with the ReAdW software (http://tools.proteomecenter.org/software.php). Protein identification was performed querying MS/MS data against the predicted proteomes of *P. yayanosii* (Uniprot Database, 2013/12/17) together with an in-house contaminant database, using the X!Tandem software (X!Tandem Cyclone (2011.12.01), http://www.thegpm.org). Proteins identified with at least two unique peptides and a log(E-value) lower than 4 were validated.

Statistical analyses to identify regulated proteins were performed with the PepC software using spectra counts for each protein^54^. Regulations between conditions were considered statistically reliable when the p-value (Student’s t-test and G-test) was below 5% and the fold change greater than 2.

### Statistical analysis

Once differentially expressed genes (multi-class SAM, TMEV software^50^) and proteins (Student’s *t*-test and G-test, PepC software^54^) were determined, over-expressed KEGG categories were identified using the KOBAS website^55^ and a binomial test. Over-represented GO terms were identified using the GO Local Exploration Map (GOLEM) software with a FDR of 5%^56^. The Enrichment Map^57^ plugin of the Cytoscape software^58^ was used to summarize the data. Syntheny of the regulated gene clusters was represented using the SyntTax software^59^ and the EasyFig software^60^.

## Additional Information

**How to cite this article**: Michoud, G. and Jebbar, M. High hydrostatic pressure adaptive strategies in an obligate piezophile *Pyrococcus yayanosii*. *Sci. Rep.* **6**, 27289; doi: 10.1038/srep27289 (2016).

## Supplementary Material

Supplementary data

Supplementary Table S3

## Figures and Tables

**Table 1:** Summary of GO and KEGG enrichment analyses in *P. yayanosii*.

Conditions	Genes		Proteins	
	DR	UR	DR	UR
20 vs 52 MPa	Hydrogenases	Chemotaxis	Ribosomes	-tRNA bisynthesis -Oxidoreductase activity
80 vs 52 MPa	Hydrogenases	Ribosomes transmembrane ATPases	-Ribosomes-Formate metabolism	-tRNA biosynthesis-Hydrogenases

The hydrostatic pressure 52 MPa is considered as the reference condition whereas 20 and 80 MPa where considered the stressful conditions. UR means up-regulated and DR means down-regulated.
