# Supplementary material for: High hydrostatic pressure adaptive strategies in an obligate piezophile Pyrococcus yayanosii
Source: Sci Rep. 2016 Jun 2;6:27289. doi: 10.1038/srep27289 (PMC4890121; doi:10.1038/srep27289)
Supplement: Supplementary data [file srep27289-s1.pdf]

High hydrostatic pressure adaptive strategies in an obligate piezophile *Pyrococcus yayanosii*

Grégoire Michoud[1,2] and Mohamed Jebbar[1*]

[1] Univ Brest, CNRS, Ifremer, UMR 6197-Laboratoire de Microbiologie des Environnements Extrêmes (LM2E), Institut Universitaire Européen de la Mer (IUEM), rue Dumont d'Urville, 29 280 Plouzané, France

[2] Current address: King Abdullah University of Science and Technology (KAUST), Biological and Environmental Sciences and Engineering Division (BESE), Thuwal 23955-6900, Kingdom of Saudi Arabia

*Corresponding author:
Prof. Mohamed Jebbar
Institut Universitaire Européen de la Mer (IUEM)
Laboratoire de Microbiologie des Environnements Extrêmes (UMR 6197)
Technopole Brest-Iroise
Rue Dumont d'Urville
29280 Plouzané
Phone: +33 298 498 817
Fax: +33 298 498 705
E-mail: mohamed.jebbar@univ-brest.fr

Supplementary Data

Table S1 General characteristics of the 4 species of *Pyrococcus* studied, *P. abyssi* GE5, *P. furiosus* DSM 3638, *P. horikoshii* OT3 and *P. yayanosii* CH1[1]

		Pyrococcus abyssi GE5	*Pyrococcus furiosus* DSM 3638	*Pyrococcus horikoshii* OT3	*Pyrococcus yayanosii* CH1
Genomic features	Size (bp)	1,768,562	1,908,256	1,738,505	1,716,818
	Predicted CDS	2,051	2,179	2,061	1,915
	GC %	45	41	42	52
	CDS %	92.86	90.41	91.94	89.91
	Mean CDS length (bp)	863	842	843	828
	rRNA	5	4	4	4
	tRNA	46	46	47	46
	arCOG %	72.94	72.28	72.84	74.57
Geographical features	Origin	Vulcano Island, Italy	North Fiji Basin, SW Pacific Ocean	Okinawa Trough, Pacific Ocean	Ashadze, Mid- Atlantic Ridge
	Depth (m)	0	2,000	1,395	4,100
Physiological features	Morphology	Cocci	Cocci	Cocci	Cocci
	Metabolism	Strictly anaerobic	Strictly anaerobic	Anaerobic	Strictly anaerobic
	Size (µm)	0.8-2	0.8-2.5	0.8-2	0.6-1.4
	Temperature range (ºC)	67-102	70-103	80-102	80-108
	Optimum temperature (ºC)	96	100	98	98
	pH range	4-8.5	5-9	5-8	6-9.5
	Optimum pH	6.8	7	7	7.5-8
	NaCl range (%, w/v)	0.7-2.5	0.5-5	1-5	2.5-5.5
	Optimum NaCl (%, w/v)	3	2	2.4	3.5
	Pressure range (MPa)	0.1-50	0.1-45	0.1-40	20-120
	Optimum Pressure (MPa)	20	0.1	0.1	52
	Doubling time (min)	33	37	32	50

Table S2 : Predicted pathways of amino acid biosynthesis. -§ is missing the last enzyme of the pathway.

Amino Acid	Predicted pathways of amino acid biosynthesis			
	P. abyssi	*P. furiosus*	*P. horikoshii*	*P. yayanosii*
Cys	+	+	-	+
Leu	-§	-§	-	-
Lys	+	+	+	-
Met	-	+	+	-
Phe	-	+	-	-
Trp	+	+	-	-
Val	-§	-§	-	-
Ala	+	+	+	+
Asn	+	+	+	+
Asp	+	+	+	+
Glu	+	+	+	+
Gln	+	+	+	+
Gly	+	+	+	+
His	-	+	-	-
Ile	-§	-§	-	-
Pro	-	-	-	-
Ser	+	+	+	+
Thr	+	+	+	+
Tyr	-	+	-	-

Table S4 Real time RT-PCR validations and operon regulation during High pressure stress. Relative gene expression changes data from microarray analysis and quantitative real-time RT-PCR assays

Locus	Description	Coordinates		Microarray		qRT-PCR	
				20-52	80-52	20-52	80-52
PYCH_00020	NiFe hydrogenase II subunit	1 016	2 029	−3,96	−3,19	−3,62	−5,52
PYCH_00030	cytochrome-c3 hydrogenase	2 016	2 882	−2,96	−2,83	−3,97	−3,89
PYCH_01430	30S ribosomal protein	132 937	133 569	2,26	3,64	1,9	2,4
PYCH_r10	ARNr 5S	164 226	164 347	−3,27	−1,15	−2,69	−1,16
PYCH_05460	hypothetical protein	452 227	452 766	2,21	−2,54	2,82	−2,15
PYCH_06240	ribulose-1,5-biphosphate synthetase	524 514	525 272	2,2	2,13	3,25	1,95
PYCH_06250	Thiamine biosynthesis protein	525 353	526 645	5,3	7,05	3,18	2,25
PYCH_08570	Transposase	755 521	756 726	−1,55	1,19	−1,05	1,24
PY0867_0868	Intergenic Space	765 083	765 369	4,5	8,91	2,35	2,45
PYCH_09590	*Cas* 4	847 106	847 621	2,23	2,96	12,34	8,94
PYCH_11050	formate hydrogenlyase II subunit	974 097	975 539	−9,25	−9,87	−12,35	−4,08
PYCH_11070	formate hydrogenlyase II subunit	976 760	978 814	−5,64	−4,94	−27,78	−3,76
PYCH_11420	NADH dehydrogenase subunit	1 004 080	1 005 255	2,08	2,38	1,75	2,11
PYCH_11930	alanine aminotransferase	1 051 537	1 051 764	1,01	1,21	1,15	1,46
PYCH_17100	maltodextrin transport - permease	1 522 525	1 523 466	2,04	3,04	1,71	1,9
PYCH_17110	maltodextrin transport - permease	1 523 463	1 524 704	1,92	2,7	1,26	2

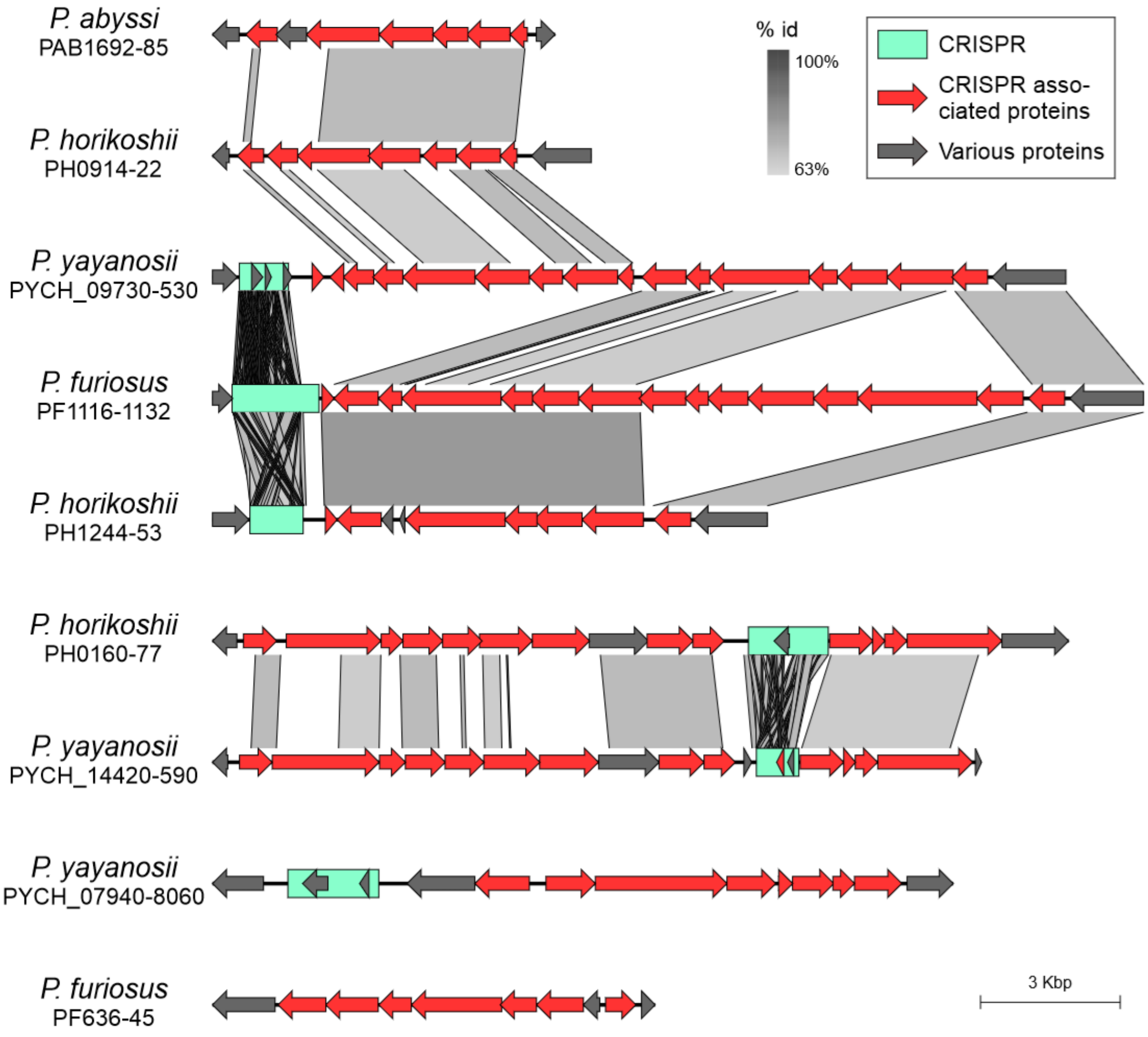

Figure S1 : CRISPR/cas alignements in Pyroccocus. The alignments were done with the Easyfig software (blastn, e-value 10^{-4})[2].

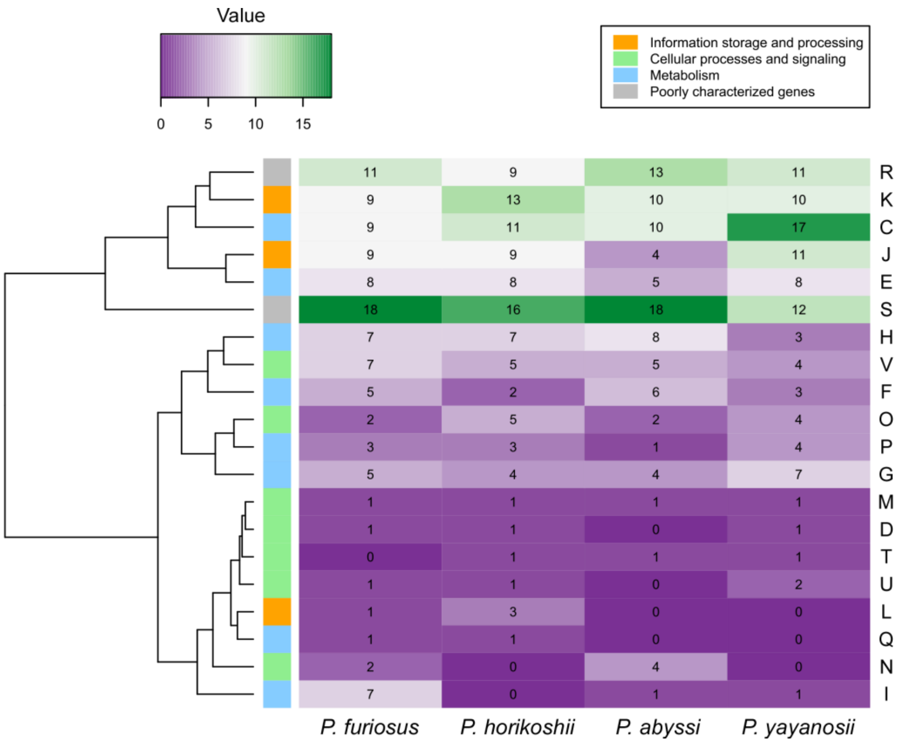

Figure S2: Heatmap representing the proportion of highly expressed genes by arCOG categories done using the R script "gplots"[3].

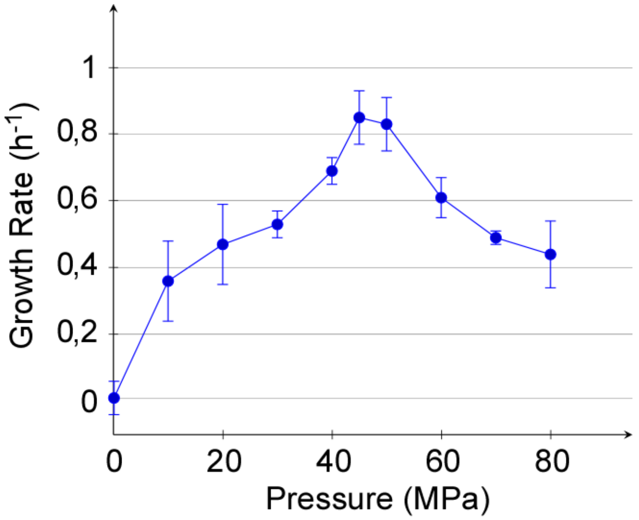

Figure S3: Growth assays of P. yayanosii.

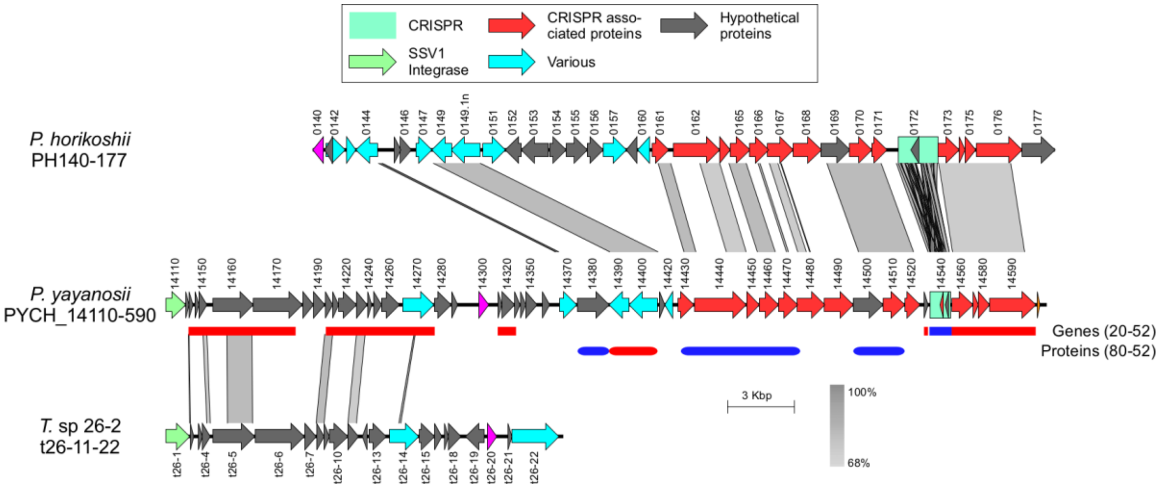

Figure S4: Alignment of a regulated *P. yayanosii* CRISPR-*cas* cluster with part of a plasmid of *Thermococcus* sp. 26-2 and *P. horikoshii* [4]. The arrows represent the genes, the ovals the expression results of RNA and the rectangle the expression results of proteins. The blue color mean the down-regulation on conditions indicated on the left whereas the red one corresponds to the up-regulation. The alignments were done with the Easyfig software (blastn, e-value 10^{-4})[2].

References

1. Birrien, J.-L. *et al. Pyrococcus yayanosii* sp. nov., an obligate piezophilic hyperthermophilic archaeon isolated from a deep-sea hydrothermal vent. *Int J Syst Evol Microbiol* **61,** 2827–2881 (2011).

2. Sullivan, M. J., Petty, N. K. & Beatson, S. A. Easyfig: a genome comparison visualizer. *Bioinformatics* **27,** 1009–1010 (2011).

3. Warnes, G. R. *et al.* gplots: Various R programming tools for plotting data. *R package version* **2,** (2009).

4. Soler, N. *et al.* Two novel families of plasmids from hyperthermophilic archaea encoding new families of replication proteins. *Nucleic Acids Res* **38,** 5088–5104 (2010).
